# Serum Leptin and Adiponectin Levels in Obese and Nonobese Asthmatic School Children in relation to Asthma Control

**DOI:** 10.1155/2013/654104

**Published:** 2013-12-17

**Authors:** Atqah Abdul Wahab, Muna M. Maarafiya, Ashraf Soliman, Noura B. M. Younes, Prem Chandra

**Affiliations:** ^1^Department of Pediatrics, Hamad Medical Corporation, P.O. Box 3050, Doha, Qatar; ^2^Weill Cornell Medical College, Doha, Qatar; ^3^Laboratory Medicine and Pathology, Hamad Medical Corporation, P.O. Box 3050, Doha, Qatar; ^4^Medical Research Center, Hamad Medical Corporation, P.O. Box 3050, Doha, Qatar

## Abstract

There is growing evidence of a positive correlation between asthma and obesity in children and adults. Leptin and adiponectin regulate several metabolic and inflammatory functions. This study aims to evaluate serum leptin and adiponectin concentrations in asthmatic school children to investigate their association with obesity and the degree of asthma control. Obese asthmatic (OA) and nonobese asthmatic (NOA) children, aged 7 to 14, were randomly enrolled in this prospective study. Data on demographic, anthropometric, serum lipids, and spirometric measures and allergy status were collected and analyzed. Serum leptin was significantly higher (25.8 ± 11.1 versus 8.7 ± 11.1; *P* < 0.0001) and adiponectin levels were lower (2.5 ± 1.2 versus 5.4 ± 2.9; *P* < 0.0001) in OA compared to NOA children. The uncontrolled group had higher leptin and lower adiponectin levels compared to well and partially controlled asthma. BMI was positively correlated with leptin (*r* = 0.79; *P* < 0.001) and negatively with adiponectin (*r* = −0.73; *P* < 0.001). Mean BMI and leptin levels were observed to be higher in girls compared to boys. Stepwise multiple linear regression analysis showed that higher BMI and female gender had significant effect on serum leptin levels. Among asthmatic children higher serum leptin and lower adiponectin levels were significantly associated with obesity and showed no significant association with degree of asthma controls.

## 1. Introduction

Several epidemiological studies have shown that the prevalence of bronchial asthma and obesity is increasing concomitantly worldwide among children and young adults [1]. The mechanisms of these pathologies remain unclear despite a number of publications on the relationship between the two diseases [2, 3]. Multiple hypotheses have been proposed. Obesity may be associated with respiratory symptoms via cardio-respiratory deconditioning, physiological restriction of the chest wall by excess adipose tissue, or comorbidities, including gastroesophageal reflux and sleep-disordered breathing [4]. The disruption of immune tolerance mechanisms by obesity-associated adipokines and cytokines has been demonstrated to be involved in the association of obesity with asthma and autoimmune diseases [5].

Obesity is characterized by chronic low-grade systemic inflammation. Obese adipose tissue is infiltrated by macrophages that are a source of inflammatory cytokines [6]. More than 50 different adipokines are secreted by adipocytes. Adipokines are proteins that help regulate various body functions [7]. Leptin and adiponectin are two adipokines that are being studied to determine their association with asthma [8]. Serum leptin is proinflammatory that affects both innate and adaptive immune responses, and serum levels are markedly increased in obesity [7, 9]. Serum adiponectin has important anti-inflammatory effects in obesity that inhibits proinflammatory cytokines (TNF-a, IL-6, and nuclear factor-kB) and induces anti-inflammatory cytokines (IL-10 and IL-1 receptor antagonist) [7, 10]. Thus, leptin and adiponectin could have a role in the pathogenesis of asthma as supported by animal studies [11, 12], and only few studies have been done in humans to convincingly establish a link between adipokines and asthma [13–15].

The aim of the present study was to evaluate serum leptin and adiponectin concentrations in asthmatic school children and to investigate their association with obesity and degree of asthma control.

## 2. Methods

### 2.1. Study Population

This prospective cohort study was conducted at the Pediatric Pulmonary Clinic of the Department of Pediatrics, Hamad Medical Corporation (HMC). From April 2012 to November 2012, 60 children (31 nonobese asthmatics and 29 obese asthmatics) met inclusion criteria. The following inclusion criteria were used: (i) age between 7 to 14 years; (ii) the diagnosis of asthma was based on physician's diagnosis and according to American Thoracic Society (ATS) guidelines [16]. In addition in accordance with guidelines of the Global Initiative with the asthmatic patients that had history of recurrent chest symptoms such as coughing, dyspnea, and wheezing, which were relieved by bronchodilator treatment, they also demonstrated reversible airflow limitation. The International Study of Asthma and Allergy in Childhood (ISAAC) questionnaire was used to assess asthma-related symptoms [17]; (iii) patients visited as followup in the pediatric pulmonary clinics at HMC. Exclusion criteria were all of the children had been free of respiratory tract infections for more than 4 weeks prior to enrollment, healthy obese and nonobese subjects with any history of chronic lung disease such as cystic fibrosis (CF), non-CF bronchiectasis, chronic lung of prematurity (BPD), interstitial lung diseases, asthma exacerbation requiring systemic steroids within 4 weeks, secondary obesity with or without association with chromosomal or genetic anomalies. This study was reviewed and approved by the local ethics committee at Hamad Medical Corporation (number 12006/12).

During their initial visits, all asthmatic patients underwent skin prick tests and serum IgE. Allergic rhinitis (AR) was assessed based on the medical history including symptoms and a physical examination, in addition to skin prick tests and Serum IgE. Patients spirometric tests were performed in the respiratory laboratory unit in accordance with standards of the American Thoracic Society [16]. The highest of three technically appropriate measurements was recorded. Forced vital capacity (FVC; in liters), forced expiratory volume in 1 second (FEV1; in liters), and FEV1/FVC were measured using a flow-sensing spirometer (Sensor Medicus Model V6200, Germany). All-age predicted values for spirometry were used and *Z*-score was calculated [18].

Anthropometric measurements were performed using digital electronic platform scale and standing height measurement without shoes using a stadiometer. Body mass index (BMI) was calculated by dividing weight in kg by height squared in meters {weight (Kg)/(height (m))^2^} and adjusted for age and gender. The standard deviation [*Z*] score for BMI was calculated. Normal weight was defined as BMI *Z*-score between −1.96 and +1.96 and obesity was defined as BMI *Z*-score >1.96. Data on demographic, anthropometric, serum lipids, spirometric measures, and allergy status were recorded and analyzed.

### 2.2. Biochemical and Hormonal Analysis

Blood samples were collected by a skilled and qualified technician at the outpatient clinic around 8 a.m. in the morning following an overnight fast. After collection, the blood samples were centrifuged for 10 minutes and stored at −70 C. The materials used for collection were disposable, adequately labeled, and of recognized quality. Leptin and adiponectin levels were measured using a commercially available enzyme-linked immunosorbent assay (ELISA) kit according to the manufacturer's instructions and standard guidelines.

### 2.3. Sample Size

The adequate sample was determined based on the primary outcome variables serum leptin and adiponectin levels between obese and nonobese asthmatic groups. As per literature review, the mean serum leptin levels in obese and nonobese asthmatic groups were found to be (16 ± 7.5 ng/mL and 8.5 ± 7.0 ng/mL) and adiponectin levels were observed to be (5.2 ± 2.5 ng/mL and 3.0 ± 2.0 ng/mL). With 90% power and at two sided 0.05 level of significance, the required sample size was 25 patients in each group. To account for the proportion of dropouts and nonparticipation it was planned to enroll 60 patients altogether.

### 2.4. Statistical Analysis

Categorical data was expressed as a frequency along with percentage and continuous data values presented in mean ± SD. Descriptive statistics were used to summarize all demographic anthropometric, serum lipids, and spirometric measures and other characteristics of the participants. Unpaired *t*-test was applied to compare mean of quantitative variables between the two independent groups (OA and NOA). Quantitative variables means between more than two independent groups (well controlled, partially controlled, and uncontrolled asthma) were analyzed using one way analysis of variance (ANOVA). The overall group was found to be significant, and Bonferroni multiple comparison test was performed for pair-wise comparison. For nonnormal or skewed data the corresponding nonparametric tests Mann-Whitney *U* and Kruskal-Wallis tests were performed. Associations between two or more qualitative variables were assessed using chi-square test. For small cell frequencies, chi-square test with continuity correction factor or Fisher's exact test was applied.

Pearson's correlation coefficient was used to assess the strength of linear relationship between two or more quantitative variables. Multiple liner regression was applied to examine and assess the effect of demographic, anthropometric, and spirometric measures and allergy status on serum leptin levels. Scatter plot was constructed and used to demonstrate the graphical presentation of the linear relationship between the two quantitative variables. A two-sided *P* value <0.05 was considered to be statistically significant. All statistical analyses were done using the statistical package SPSS 19.0 (SPSS Inc., Chicago, IL).

## 3. Results

Between the periods from April 2012 to November 2012, a total of 60 asthmatic children, 29 with obesity and 31 without obesity, were enrolled in the study at the Pediatric Pulmonary Clinic, HMC. There were 14 (23.3%) girls and 46 (76.7%) boys and mean age in obese asthmatic children was 10.9 ± 1.9 years compared to nonobese asthmatic children 11.3 ± 2.2 years (*P* = 0.432). The percentage of patients with allergic rhinitis was higher in obese asthmatic children compared to nonobese asthmatic children (62.1% versus 48.4%; *P* = 0.287). The demographic, anthropometric, serum lipids, and spirometric measures, allergy status, and other characteristics of the study population are shown in Table 1. There were no significant differences between OA and NOA regarding age, sex, atopy serum total IgE, FEV1 *Z*-score, and FVC *Z*-score (*P* > 0.05).

As expected, serum leptin levels were significantly higher among OA children compared to NOA children (25.8 ± 11.1 ng/mL versus 8.7 ± 11.1 ng/mL; *P* < 0.0001) while serum adiponectin levels were significantly lower among OA children compared to NOA children (2.5 ± 1.2 ng/mL versus 5.4 ± 2.9 ng/mL; *P* < 0.0001). Mean steroid dose does not differ significantly between obese and nonobese asthmatic children (301.1 ± 152.8 versus 299.7 ± 180.1; *P* = 0.975) as presented in Table 1. Most of the patients were considered to have adequate asthma controls (53.3% well controlled and 40% partially controlled) and 6.7% uncontrolled asthma with their respective medications. Serum leptin levels in well controlled, partially controlled, and uncontrolled asthma groups were found to be 17.01 ± 14 ng/mL, 16.04 ± 14.52 ng/mL, and 22.25 ± 12.37 ng/mL, respectively. The serum leptin concentration in the uncontrolled group was higher than that in the well and partly controlled, but the difference was not statistically significant (*P* = 0.719). The serum adiponectin concentration in the uncontrolled group was observed to be lower 2.3 ± 1.2 ng/mL compared to well controlled 3.8 ± 2.5 and partially controlled 4.6 ± 2.9 groups; however it did not differ significantly (*P* = 0.236). It is worth noting that the obesity was found to be similar across the asthma control groups. Lung function tests including FEV1 *Z*-score and FEV1/FVC *Z*-score ratio were strongly associated with degree of asthma controls. Mean FEV1/FVC *Z*-score was observationally higher among well controlled asthma −0.64 ± 0.81 compared to partially controlled −1.13 ± 0.84 and uncontrolled asthma −1.21 ± 0.83 (*P* = 0.099). No significant association was observed between age, gender, BMI *Z*-score, rhinitis, serum total IgE, and asthma controlled groups. The comparison in the serum leptin, adiponectin concentrations, and other parameters in the three groups of asthma control is shown in Table 2.

**Figure 1:**
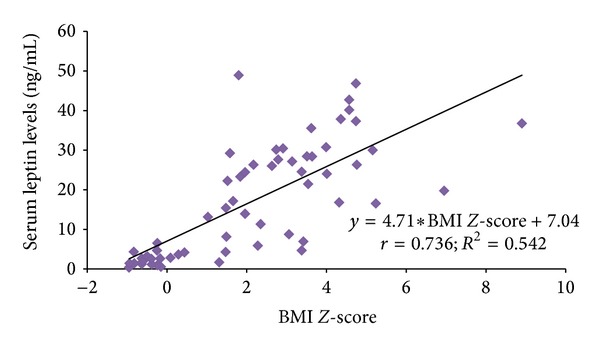
Relationship between serum leptin levels and BMI *Z*-score.

**Figure 2:**
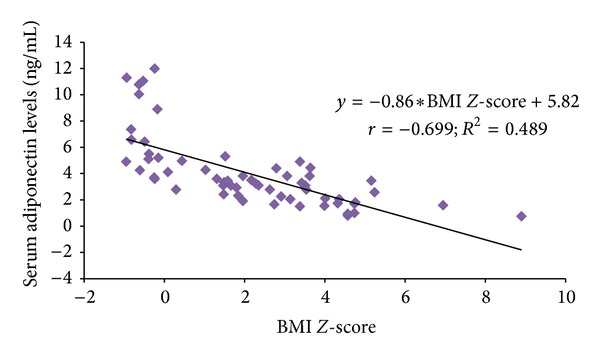
Relationship between serum adiponectin levels and BMI *Z*-Score.

For the whole group of patients, BMI *Z*-score was positively correlated with serum leptin levels (*r* = 0.74; *P* < 0.001) and negatively correlated with adiponectin levels (*r* = −0.70, *P* < 0.001) as demonstrated in Figures 1 and 2. The leptin level showed a significant negative correlation with the adiponectin levels (*r* = −0.61; *P* < 0.001). No significant correlation was observed between leptin levels, IgE, and spirometric parameters (data not shown here). Mean BMI and leptin were found to be higher in girls (24.6 ± 5.9 ng/mL versus 23.7 ± 7.2 ng/mL; *P* = 0.687) and (22.7 ± 14.2 ng/mL versus 15.2 ± 13.4 ng/mL; *P* = 0.081) compared to boys whereas mean adiponectin levels were significantly lower among girls compared to boys (2.6 ± 1.1 ng/mL versus 4.4 ± 2.8 ng/mL; *P* = 0.028). Stepwise multiple linear regression analysis showed that higher BMI (regression coefficient *β* = 1.59; *t* = 10.3; *P* < 0.001) and female gender (regression coefficient *β* = 6.02; *t* = 2.41; *P* = 0.019) have had significant effect on serum leptin levels accounting effect of other covariates such as age, rhinitis, IgE, FEV1 *Z*-score, and FVC *Z*-score.

## 4. Discussion

There has been intense interest in the potential role of adipose tissue in the development of asthma in obesity and in the pathogenesis of asthma. Whether activity restriction causes obesity or obesity by itself causes the development of asthma has been questioned. The possibility that asthma may lead to obesity is less controversial, because of fear of exercise or inability to exercise regularly. The adipose tissue in obese subjects leads to a systemic inflammatory state which produces a rise in the serum concentrations of several proinflammatory cytokines, chemokines, and adipokines, such as leptin as proinflammatory and adiponectin as anti-inflammatory. As body weight increases, more leptin is produced as expected in our study population, where serum leptin levels show positive correlation with BMI *Z*-Score with higher levels in OA children in our study population. This is in agreement with the previous studies [19, 20]. Adiponectin demonstrated a strong negative correlation with BMI *Z*-Score with lower levels in OA children, in contrast to a recent study where serum leptin levels were not elevated in obese asthmatics compared to nonobese asthmatics or controls but were only significantly raised in obese children without asthma [21]. Although the relationship among obesity, asthma, and leptin cannot be adequately addressed in this study, we found that BMI is determining factor for leptin; however it did not have a significant association with asthma controls.

The role of adipokines quite likely varies between different asthma inflammatory processes and reported data are inconclusive regarding the independent association between serum leptin or adiponectin and the risk of asthma. Adipocytes in the white adipose tissue are the main source of leptin, but adipocytes also secrete cytokines like TNF-*α*, IL-6, and IL-10. TNF-*α* stimulates the production of Th2 type cytokines. In summary, a common inflammatory pathway in both, obesity and asthma, is orchestrated by TNF. Similar to leptin human data on the independent association between serum adiponectin concentration and asthma are currently inconclusive [22]. In a recent study there were no significant differences between cell counts in induced sputum (eosinophils, macrophages, lymphocytes, and neutrophils) between obese and nonobese asthmatic patients [23]. Asthma is often considered as a single disease entity, but it is actually a syndrome with many different pathological pathways ultimately leading to quite similar clinical presentation: variable airway obstruction with chest tightness, wheezing, and cough [24]. Previously, conflicting results on the levels of adipokines in patients with asthma have been published including childhood asthma. Leptin has been reported to be increased [14, 25] or normal [26, 27] and adiponectin either decreased [28, 29] or normal [26, 27]. The conflicting results are likely explained by differences in patient selection. In addition, there are patient-related contributing factors like age, sex, and fat distribution. In this study, we did not find any correlation between leptin levels and IgE or spirometric parameters although FEV1 is considered a measure of asthma severity; it is not consistently related to inflammation or symptoms and is affected by multiple factors.

This study examined the association of serum leptin and adiponectin in OA and NOA children in relation with asthma control and we found that serum leptin and adiponectin concentrations did not differ among well controlled, partial controlled, and uncontrolled asthma groups suggesting that serum leptin levels may not have a major role among asthma control groups with obesity. In a recent study, where serum leptin levels were compared in intermittent, mild persistent, and moderate persistent asthma groups and found an increase in leptin levels correlated with the increase in the clinic severity of asthma, suggesting that serum leptin levels may reflect clinic severity of asthma, BMI *Z*-score also did not differ significantly among the asthma control groups. These findings were supported by Stanford et al. that enrolled 2238 outpatient adults and 2429 children across the United States and found that high BMI was not a predictor of controlled asthma in children (OR 1.54) [30].

We found that in obese and nonobese female asthmatic children, the levels of leptin were higher and adiponectin levels were lower than boys. The influence of leptin on increasing asthma risks varied by gender has been reported [31]. Among asthmatic children, higher serum leptin levels were shown to have stronger associations with female gender [32]. On the other hand, in a case control study conducted in 102 prepubertal asthmatic children, significant difference was observed in serum leptin levels between asthmatic and healthy children. However, this difference was confined entirely to boys [25]. Our study obviously has some limitations. First, small percentages of the subjects enrolled were girls. To our knowledge this is the first study of this kind to report leptin and adiponectin levels in OA and NOA children in relation to the degree of asthma control. Though, we have small percentage of patients in the uncontrolled asthma group and our data did not demonstrate a significant association between obesity and the degree of asthma control. This can be explored and validated in another larger study.

## 5. Conclusion

In conclusion, our study findings indicate that among asthmatic school children higher serum leptin and lower adiponectin levels were significantly associated with obesity. Serum leptin and adiponectin levels did not show significant effect on the degree of asthma controls. Other factors should be sought for a better understanding of the connection between serum leptin and adiponectin levels with obesity and asthma controls. Further, larger prospective study with adequate statistical power needs to be conducted for generalization of the above findings.

## Figures and Tables

**Table 1:** Patients' demographic anthropometric, serum lipids, and spirometric measures between obese and nonobese asthmatics school children.

Variables	Obese asthmatic *N* = 29 (48.3%)	Nonobese asthmatic *N* = 31 (51.7%)	*P* value
Age (years)	10.9 ± 1.9 (10.7; 7.5–14.0)	11.3 ± 2.2 (11.2; 7–14)	0.432
Gender			
Male	22 (75.9%)	24 (77.4%)	0.887
Female	7 (24.1%)	7 (22.6%)	
BMI *Z*-score	3.9 ± 1.4 (3.6; 2.2–8.9)	0.38 ± 1.04 (−0.15; −0.95–1.96)	<0.0001
Regular daily inhaled corticosteroid (yes)	22 (75.9%)	23 (74.2%)	0.881
Steroid dose (*µ*g/day)	301.1 ± 152.8 (250; 100–640)	299.7 ± 180.1 (250; 100–640)	0.975
Asthma control			
Well controlled	15 (48.4%)	17 (58.6%)	0.696
Partially controlled	14 (45.2%)	10 (34.5%)	
Uncontrolled	2 (6.5%)	2 (6.9%)	
Allergic rhinitis (yes)	18 (62.1%)	15 (48.4%)	0.287
Serum IgE	537.4 ± 545.4 (381; 15–2264)	445.6 ± 490.3 (208; 6–1663)	0.495
FEV1 *Z*-score	−0.77 ± 0.88 (−0.70; −2.3–1.1)	0.99 ± 1.0 (−0.89; −3.7–1.1)	0.399
FVC *Z*-score	−0.29 ± 0.77 (−0.09; −1.7–1.8)	−0.37 ± 1.13 (−0.45; −2.9–2.3)	0.775
FEV1/FVC *Z*-score	−0.87 ± 0.75 (−1.0; −2.5–0.67)	−0.88 ± 0.95 (−0.95; −2.5–0.69)	0.929
Serum leptin (ng/mL)	25.8 ± 11.1 (27.1; 4.6–46.8)	8.8 ± 11.1 (3.6; 0.32–48.9)	<0.0001
Serum adiponectin (ng/mL)	2.5 ± 1.2 (2.3; 0.75–4.9)	5.4 ± 2.9 (4.3; 1.9–12.0)	<0.0001

Values in parenthesis denote median and range.

**Table 2:** Association of demographic anthropometric, serum lipids, and spirometric measures with degree of asthma controls.

Variables	Asthma control			*P* value
	Well controlled *N* = 32 (53.3%)	Partially controlled *N* = 24 (40%)	Uncontrolled *N* = 4 (6.7%)	
Age (years)	10.75 ± 1.9	11.3 ± 2.3	12.5 ± 1.4	0.218
Gender				
Male	22 (68.7%)	22 (91.7%)	2 (50%)	0.181
Female	10 (31.3%)	2 (8.3%)	2 (50%)	
BMI *Z*-score ≤ 1.96	15 (46.9%)	14 (58.3%)	2 (50%)	0.769
BMI *Z*-score > 1.96	17 (53.1%)	10 (41.7%)	2 (50%)	
Regular daily inhaled corticosteroid (yes)	31 (96.9%)	12 (50%)	2 (50%)	<0.001
Allergic rhinitis (yes)	13 (40.6%)	17 (70.8%)	3 (75%)	0.132
Serum IgE	393.8 ± 529.8	618 ± 504.9	489.3 ± 369.9	0.208
FEV1 *Z*-score	−0.58 ± 0.89	−1.28 ± 1.03	−0.86 ± 0.86	0.052
FVC *Z*-score	−0.24 ± 0.86	−0.51 ± 1.04	0.08 ± 1.21	0.684
FEV1/FVC *Z*-score	−0.64 ± 0.81	−1.13 ± 0.84	−1.21 ± 0.83	0.099
Serum leptin (ng/mL)	17.01 ± 14.0	16.04 ± 14.5	22.25 ± 12.4	0.719
Serum adiponectin (ng/mL)	3.78 ± 2.5	4.60 ± 2.9	2.31 ± 1.2	0.236
